# Validity and reliability of the VOAA-DDD to assess spontaneous hand use with a video observation tool in children with spastic unilateral cerebral palsy

**DOI:** 10.1186/1471-2474-10-145

**Published:** 2009-11-25

**Authors:** Pauline BM Aarts, Peter H Jongerius, Yvonne A Geerdink, Alexander C Geurts

**Affiliations:** 1 Department of Pediatric Rehabilitation, Sint Maartenskliniek, Hengstdal 3,6522 JV Nijmegen, the Netherlands; 2 Department of Rehabilitation, Radboud University Nijmegen - Medical Centre, Nijmegen, the Netherlands

## Abstract

**Background:**

In 2003 new computer software, the VOAA (Video Observations Aarts and Aarts), was designed to score and evaluate two important aspects of spontaneous upper limb use, i.e. overall duration and frequency of specific behaviours. The aim of this study was to investigate the test-retest, interrater and intrarater reliability and the construct validity of a new module, the VOAA-DDD, to determine developmental disregard in children with spastic unilateral cerebral palsy (CP).

**Methods:**

A test-retest design with three raters for reliability and a two-group design for construct validity were used. Subjects were a total of 20 children with spastic unilateral CP equally divided in two age groups (2.5-5 and 5-8 years), and 56 healthy children of the same age groups. Overall duration and frequency of specific behaviours of the affected arm and hand were assessed during a task demanding ('stringing beads') and a task stimulating ('decorating a muffin') the use of both hands. Reliability was estimated by intraclass correlation coefficients (ICCs). Construct validity was assessed by comparing children with CP to healthy children.

**Results:**

All ICCs exceeded 0.87. In contrast with healthy children, children with CP used their affected hand less during the 'muffin' task compared to the 'beads' task. Of the children with CP, 90% in the age group of 2.5-5 years and 50% in the age group of 5-8 years showed values exceeding the extreme values of healthy controls, respectively, indicating developmental disregard.

**Conclusion:**

The VOAA-DDD is a reliable and valid instrument to assess spontaneous use of the affected arm and hand in order to determine developmental disregard in children with spastic unilateral CP.

## Background

Cerebral Palsy (CP) is defined as a non-progressive clinical syndrome with movement and postural impairments due to brain damage before the age of 1 year and has an incidence of approximately 2.4 per 1000 live births[1]. The resulting movement impairments are frequently lateralized, usually with serious involvement of the upper extremity. In particular, fine motor control of the hand and fingers is compromised. The involved extremity may demonstrate disorders in muscle tone, dyskinetic and atactic motor disorders as well as a reduction in strength and sensibility. Consequently, the paretic extremity may be used below the level of individual capacity in a broad range of age-appropriate tasks. Indeed, several converging lines of evidence suggest that non-use of a deafferented limb in monkeys or of the paretic arm in patients with unilateral stroke or CP is a learning phenomenon leading to a conditioned suppression of movement on the affected side, which is referred to as 'learned non-use' in adults and 'developmental disregard' in children[2,3]. Developmental disregard is not so much reflected in the individual capacity of the child to involve the affected limb in tasks that require bimanual performance, but rather in the overall duration of use and the frequencies of specific behaviours of the affected arm and hand during tasks that allow predominantly single-handed performance. Very often, children with unilateral CP will tend to limit the use of their affected hand to simple functions, for example as a 'stabilizer' for the non-affected hand[4]. In this perspective, many young children with an asymmetric upper limb function due to CP must be stimulated to improve their bimanual performance, especially in their pre-school and primary school age[5].

In previous research[6-8] the use of the paretic hand in children with CP has been assessed by videotaping bilateral manipulation. Yet, the applied procedures have not been described and tested in detail. As a result widespread replication and implementation of these methods would not be justified. More importantly, many available instruments focus on the individual capacity of arm and hand movements during tasks demanding optimal use, whereas the amount of spontaneous use of the affected limb (in terms of overall duration and frequency of specific behaviours) is not assessed. For example, the Melbourne Assessment of Unilateral Upper Limb Function (Melbourne)[9] comprises 16 different skills among which range of motion, target accuracy, and fluency of movement are scored, and the Quality of Upper Extremity Skills Test (QUEST)[10] scores grasping, weight-bearing and protective extension as important skills. Only the Assisting Hand Assessment (AHA)[11,12] has been described and evaluated in detail. It consists of 22 items that focus on the effectiveness of use of the assisting hand during bimanual activities in a semi-structured play situation and summarizes all behaviours into one sum score. Yet, none of the existing tests specifically focuses on the amount of spontaneous use of the affected limb during 'natural' activities as the most important indicator of developmental disregard. Indeed, existing instruments do not quantify how often and for how long the affected limb is used, and which variety of behaviours is shown, during different types of tasks.

In 2003 new computer software, the VOAA (Video Observations Aarts and Aarts), was designed to score and evaluate two important aspects of spontaneous upper limb use, i.e. overall duration and frequency of specific behaviours during predetermined, age-appropriate activities[5]. The scoring system of the VOAA-software supports the raters with well standardized videos that can be accurately and reliably assessed using buttons for all pre-determined behaviours that are linked to an integrated timer function. Furthermore, the software allows fast descriptive and statistical analyses. The applied module, which consisted of three tasks (making a sandwich, building Lego blocks and taking off shoes), showed an excellent content validity index (0.93) and a good intrarater and interrater reliability (Cohen's kappa 0.62-0.85). However, in order to identify developmental disregard, it was necessary to develop a new module (VOAA-DDD) with two well designed tasks with greater subtask variety and a better contrast between the tasks in terms of the necessity to use the affected arm and hand: 1) 'stringing beads' *demanding *the use of both hands, and 2) 'decorating a muffin' *stimulating *the use of both hands. These tasks were standardized and adjusted for two age groups: 2.5-5 years and 5-8 years.

Because in the VOAA-DDD two new tasks are introduced with a specific diagnostic purpose, the goal of the present study was to provide insight into some important properties of the VOAA-DDD. In this perspective, the following research questions were posed:

1. What is the interrater, intrarater and test-retest reliability of the VOAA-DDD in children with spatic unilateral CP between 2.5 and 8 years of age?

2. Is the VOAA-DDD a discriminative instrument to distinguish children with spastic unilateral CP in the age of 2.5 and 8 years from healthy children of the same age groups?

The latter question was aimed to underscore the construct validity of the VOAA-DDD. It was hypothesized that in healthy children the overall duration and frequency of specific hand use would be equal for both hands and the same in either task (stringing beads or decorating muffin), whereas children with CP would be more inclined to disregard their affected arm and hand, particularly during the task that merely stimulated the use of the impaired limb (decorating a muffin). The duration of hand use was considered the most sensitive and primary outcome measure for identifying developmental disregard.

## Methods

### Participants

Twenty children with spastic unilateral CP were tested with the Melbourne and the VOAA. The children were recruited from 5 rehabilitation centres and affiliated schools. Ten children were between 2.5-5 years (mean age 3.4 ± 0.8; age group 1) and 10 between 5-8 years of age (mean age 6.8 ± 0.7; age group 2). The demographic and functional characteristics of these children are summarized in Table 1. In addition, 56 healthy children were tested with the VOAA. They were recruited from regular schools and divided in the same age groups: 2.5-5 years (n = 26) and 5-8 years (n = 30). The study was approved by the regional Medical Ethical Committee on Research Involving Human Subjects (CMO nr. 2006/194). In all cases, oral and written informed consent was obtained from the legal caregivers.

**Table 1:** Group characteristics of the children with CP

Characteristics		Group 1	Group2
Age	mean ± SD	3.4 ± 0.77	6.8 ± 0.66
	
	range	2.5 - 4.9	5.7 - 7.8

Affected side	right	4	7
	
	left	6	3

Gender	male	3	8
	
	female	7	2

(Pre)school type	regular	9	3
	
	special	1	7

MACS	I	4	5
	
	II	5	4
	
	III	1	1

Melbourne	mean ± SD	56.9 ± 16.7	61.1 ± 18.3
	
	range	30 - 80	32 - 85

### Raters

To assess the children with CP, three occupational therapists were selected who had a broad experience in training the target group. Two occupational therapy students assessed the healthy children. All received a 3-hour training for scoring the VOAA-DDD. None of the raters was familiar with any of the children.

### Tasks

**Figure 1:**
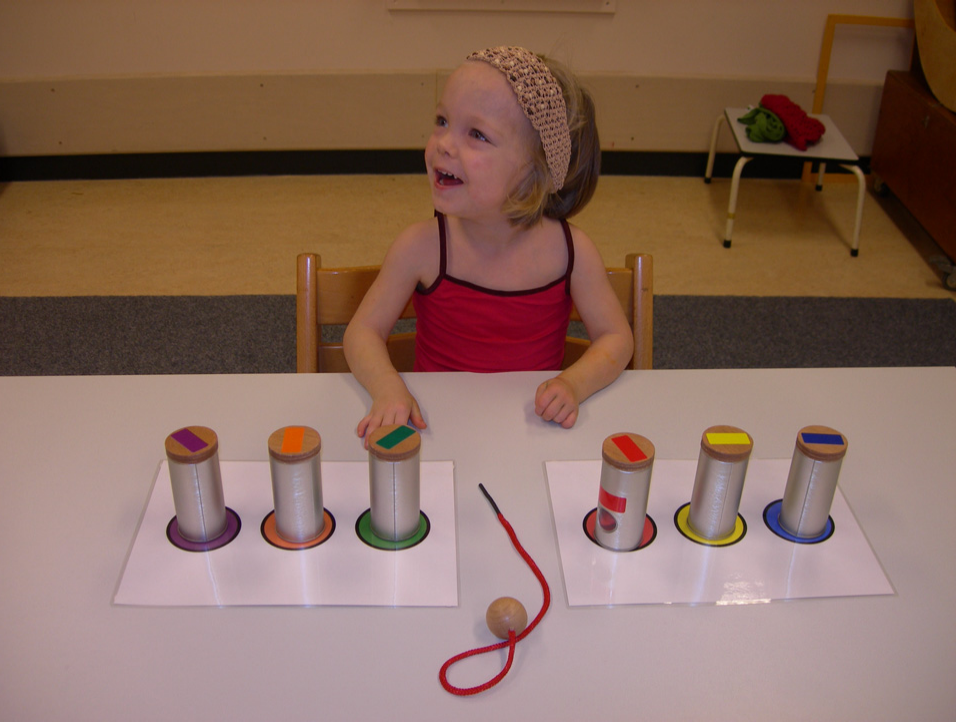
**A two-years old child stringing beads**.

**Figure 2:**
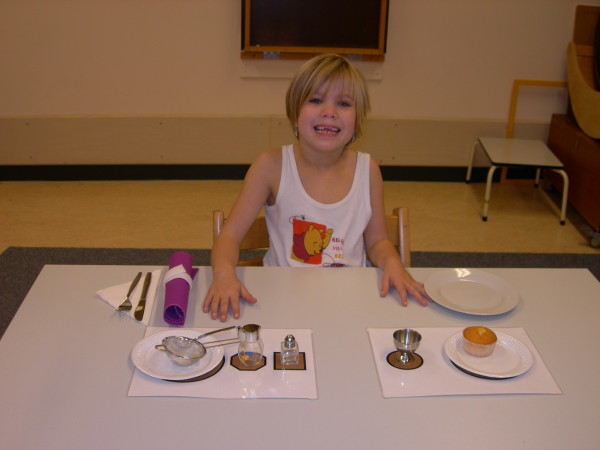
**A five-years old child decorating a muffin**.

To investigate the amount of spontaneous use of the affected arm in bimanual activities two standardized tasks were designed, of which one had to stimulate and the other had to demand the use of the paretic arm and hand. Other prerequisites were that both tasks had to be attractive, be age appropriate, and have a repetitive character with a maximum performance time of about 5 minutes. 'Stringing beads' was selected as the task *demanding *the use of both hands, which would reflect the individual child's capacity. The utensils were chosen and displayed in such a way that it would be fairly impossible not to use the affected arm and hand in any subtask. A child was asked to take beads from each of six different cans and string them on a thread (Figure 1). Taking and stringing a bead from each can was considered a subtask. In this way six similar subtasks had to be performed with only slightly different positions of the cans. 'Decorating a muffin' was selected as the task stimulating the use of both hands, which would reflect the individual child's 'natural' performance. The utensils were chosen and displayed to provoke the use of both arms, but most subtasks could still be executed one-handed. First, a child (Figure 2) was asked to put a placemat on the table and to put a plate on the placemat (subtask 1). Then, the child was asked to take the muffin from the saucer and put it on the plate, and to take the paper off the muffin and put the paper back on the saucer (subtask 2). The child was asked to subsequently decorate the muffin by taking candy's from the egg-cup (subtask 3) and two pots (subtasks 4 and 5) and to put the caster sugar on the muffin (subtask 6). When the decoration of the muffin was finished, the older children were asked to cut it into pieces or to take a piece of the muffin for the younger children (subtask 7) and to end the task by cleaning the hands with a napkin (subtask 8). Age group 1 was not required to use the placemat and the cutlery. This difference resulted in seven subtasks for age group 1 and eight subtasks for age group 2.

During both tasks, children sat on a chair with both their hands supported on the table and both feet supported on a footplate. The test instructor sat opposite to the child. The view angle of the camera is diagonally facing the affected side of the child in the Figures 1 and 2 for the beads and muffin task, respectively. The healthy children were all recorded from a frontal view. The test instructor informed the child what was expected in terms of utensils and results ('a string of beads' and 'a decorated muffin') but did not give any clue as to what type of motor performance was expected. During the task, the instructor merely pointed at the materials of the forthcoming subtask, working from the affected to the non-affected body side. In this way, all children were able to understand the goals of both tasks.

### Scoring system

All videos were digitally stored and linked to a computerized scoring program with an integrated timer. Thus, each behaviour could easily be scored in terms of frequency (how often) and duration (how long). The videos were evaluated by the raters off line. The scores were incorporated in the database containing the basic information about the child and the videos. Table 2 shows the essential features of the VOAA analysis. First, a duration score was determined for both tasks, reflecting the percentage of the overall time of spontaneous use of the affected arm and hand during either task (ranging from 0 to 100%). The duration score was considered the primary outcome for identifying developmental disregard. In addition, the occurrence of 10 pre-defined motor behaviours was scored. Three frequency scores were determined based on the observation of specific motor behaviours of the affected arm and hand: a basic, an advanced, and a variation score. The three behaviours Grasps, Holds and Releases were observed in a basic form during the muffin and the beads task (see note Table 2). In addition, these behaviours were scored in an advanced form for the beads task only. One point could be obtained for each behaviour in each of the subtasks. The raw sum score for the muffin task could, thus, vary from 0 to 21 in the young group (7 subtasks * 3 behaviours) and from 0 to 24 in the older age group (8 subtasks * 3 behaviours) (B-score). The raw sum score for the beads task could vary from 0 to 18 (6 subtasks * 3 behaviours) in both age groups (B- and A-score). Lastly, a raw variation sum score was given representing the number of all possible behaviours (see Table 2) registered during any tasks, varying from 0 to 10 (all 10 behaviours) (V-score). Because of the different numbers of subtasks, all frequency scores (both the sum scores and the individual behaviours) were converted to percentage scores, ranging from 0 to 100%. For example, a percentage B-score of 50% indicated that a child showed the specific behaviours grasps, holds and releases in 50% of the possible subtasks. The percentage B-, A- and V-scores were used for statistical analysis.

**Table 2:** Features of the VOAA-DDD beads and muffin tasks

	Duration Scores	Frequency scores
Objective	To determine the percentage of spontaneous use of the affected arm and hand in a fixed time period	To determine the occurrence of specific motor behaviours of the affected arm and hand during different subtasks

Time-related	Yes	No

Observed motor	Use of the affected arm and hand	Reaches
behaviour	No use of the affected arm and hand	Grasps*
		Holds*
		Releases*
		Puts back on place
		Stabilizes
		Shakes/pours
		Pushes/pulls/shoves/rubs
		Catches/carries
		Manipulates

Scores	Duration beads: total % of use of the affected arm and hand related to the time for completing the beads task	B-score: total of basic Grasps, Holds and Releases observations during the tasks
		A-score: total of advanced Grasps, Holds and Releases observation during the beads task
		V-score: total of the following behaviours during both tasks:
	Duration muffin: total % of use of the affected arm and hand related to the time for completing the muffin task	1. Stabilizes, 2. Reaches, 3. Puts back on place, 4. Shakes/pours, 5. Pushes/pulls/shoves/rubs, 6. Catches/carries, 7. Manipulates, 8. advanced Grasps, 9. advanced Holds and 10. advanced Releases

* With regard to the behaviours Grasps, Holds, and Releases we used both a basic (B) and an advanced (A) description for the beads task; for the muffin task we used only a basic (B) score. Criteria are related to the quality of the performance; for all active behaviours a B-score is obtained, but for higher qualitative grasping, holding and releasing an A-score can be gained. E.g. all active releasing is a B-score, but releasing with wrist extension gives an A-score.

### Procedure

Each child was examined twice by executing both tasks of the VOAA-DDD with a time interval of two weeks between the sessions. All first assessments were scored twice by the three raters with a minimum time interval of two weeks. These data were used to determine the intrarater and interrater reliability. The second assessments were only scored by one rater and were used to establish the test-retest reliability. Each healthy child was tested and scored once with the VOAA-DDD by one of the two raters. In healthy children the duration and percentage scores were determined for the non-dominant arm and hand. All raters were permitted to view the videotapes in slow motion or freeze-frame and to rate them as many times as they deemed necessary.

### Statistics

Interrater, intrarater and test-retest reliability were calculated by using Intraclass Correlation Coefficients (ICCs), validated for use by multiple raters, and evaluated in a two-way random model for absolute agreement. As for the percentage scores, the A-score of the beads task, the B-score of the muffin task and the V-score were used to calculate ICCs. For the evaluation of the agreement, the classification of Landis and Koch[13] was used: ICC 0.01-0.20 = slight agreement; 0.21-0.4 = fair; 0.41-0.60 = moderate; 0.61-0.80 = substantial; 0.81-1.0 = almost perfect agreement.

Descriptives were used to reflect the duration and percentage scores for the affected arm and hand in children with CP and for the non-dominant arm and hand in healthy children. To test whether differences in duration scores between the beads and the muffin tasks were larger in the children with CP than in healthy children, an independent samples T-test was used. To identify developmental disregard in individual children with CP, the extreme values of the differences in the duration scores between the beads and the muffin tasks in each group of healthy children (i.e. the worst individual performances in each age group) were used as a reference. As for the percentage scores, only the B-scores of both tasks were compared to determine construct validity. To test whether differences in percentage scores between the beads and the muffin tasks were larger in the children with CP than in healthy children, a Mann Whitney U test was used.

## Results

### Reliability

Tables 3 and 4 indicate that the interrater and intrarater reliability of all three percentage scores ranged from 0.95 to 1.00 in both age groups. The interrater and intrarater reliability of the duration scores was 1.00 for both age groups. The test-retest reliability of both the duration and percentage scores varied from 0.87 - 0.99 in both age groups (Tables 3 and 5).

**Table 3:** Interrater reliability and test-retest reliability of the percentage frequency scores in the children with CP

	Interrater reliability		Test-retest reliability	
	**Group 1, n = 10**	**Group 2, n = 10**	**Group 1, n = 10**	**Group 2, n = 10**
	**Age 2.5-5 years**	**Age 5-8 years**	**Age 2.5-5 years**	**Age 5-8 years**
	**ICC (95% CI)**	**ICC (95% CI)**	**ICC (95% CI)**	**ICC (95% CI)**

B-score^1^	0.99 (0.97-1.00)	1.00 (0.99-1.00)	0.97 (0.89-0.99)	0.99 (0.95-1.00)

A-score^2^	0.97 (0.90-0.99)	0.95 (0.86-0.99)	0.87 (0.42-0.97)	0.90 (0.58-0.98)

V-score^3^	0.97 (0.92-0.99)	0.99 (0.98-1.00)	0.88 (0.55-0.97)	0.98 (0.90-0.99)

^1^obtained from the muffin task (basic Grasps, Holds and Releases observations)

^2 ^obtained from the beads task (advanced Grasps, Holds and Releases observations)

^3 ^obtained from both tasks (Stabilizes, Reaches, Puts back on place, Shakes/pours, Pushes/pulls/shoves/rubs, Catches/carries, Manipulates, and advanced Grasps, Holds and Releases observations)

**Table 4:** Intrarater reliability of the percentage frequency scores in the children with CP

	Group 1, n = 10 Age 2.5-5 years ICC (95% CI)	Group 2, n = 10 Age 5-8 years ICC (95% CI)
B-score^1 ^rater 1	1.00 (0.98-1.00)	1.00 (1.00-1.00)

A-score^2 ^rater 1	0.99 (0.98-1.00)	0.96 (0.83-0.99)

V-score^3 ^rater 1	0.98 (0.91-0.99)	0.97 (0.88-0.99)

B-score^1 ^rater 2	1.00 (0.99-1.00)	1.00 (0.99-1.00)

A-score^2 ^rater 2	0.97 (0.87-0.99)	0.97 (0.86-0.99)

V-score^3 ^rater 2	0.98 (0.92-1.00)	0.97 (0.84-0.99)

B-score^1 ^rater 3	1.00 (1.00-1.00)	1.00 (0.99-1.00)

A-score^2 ^rater 3	0.99 (0.97-1.00)	0.99 (0.97-1.00)

V-score^3 ^rater 3	0.99 (0.98-1.00)	1.00 (0.99-1.00)

^1^obtained from the muffin task (basic Grasps, Holds and Releases observations)

^2 ^obtained from the beads task (advanced Grasps, Holds and Releases observations)

^3 ^obtained from both tasks (Stabilizes, Reaches, Puts back on place, Shakes/pours, Pushes/pulls/shoves/rubs, Catches/carries, Manipulates, and advanced Grasps, Holds and Releases observations)

### Construct validity

**Figure 3:**
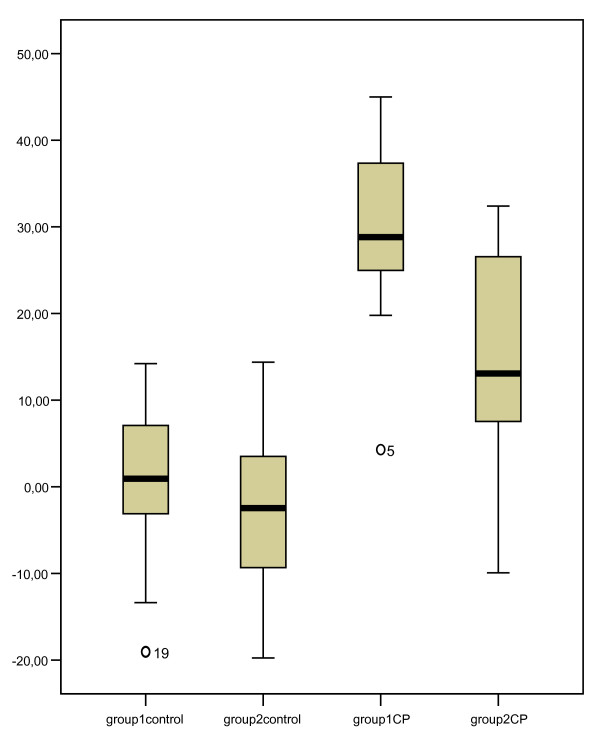
**Box plots of the differences in duration scores**. Beads vs. muffin for both the children with CP and the healthy children; group 1 = age group 2.5-5 years; group 2 = age group 5-8 years.

Table 5 shows the mean duration scores for both the children with CP and the healthy children. In contrast to the healthy children, the children with CP clearly used their affected arm and hand less during the muffin task compared to the beads task (age group 1, mean difference: 29.6 ± 12.2%, t = -8.1, p < 0.001; age group 2, mean difference: 13.5 ± 13.7%, t = -4.6, p < 0.001). To identify individual children with developmental disregard, the extreme values of the differences in duration scores between the muffin and the beads task in the healthy children were used as a reference. Figure 3 shows that the extreme values of the healthy children were 14.2 and 14.4 for age groups 1 and 2, respectively. Ninety percent of the children with CP in age group 1 and 50% of the children with CP in age group 2 showed greater differences in duration scores than 14.2 and 14.4, respectively, and could be identified as suffering from developmental disregard.

**Table 5:** Duration scores for healthy children and children with CP as well as test-retest reliability of the duration scores for children with CP

	Mean Duration		Test-retest reliability	
	**Group 1, n = 10** **Age 2.5-5 years** **% (SD)**	**Group 2, n = 10** **Age 5-8 years** **% (SD)**	**Group 1, n = 10** **Age 2.5-5 years** **ICC (95% CI)**	**Group 2, n = 10** **Age 5-8 years** **ICC (95% CI)**

Muffin CP	28.7 ± 16.9	46.6 ± 26.1	0.87 (0.49-0.97)	0.97 (0.88-0.99)

Beads CP	58.3 ± 22.4	60.1 ± 25.9	0.98 (0.91-0.99)	0.99 (0.95-1.00)

Muffin Control	79.4 ± 7.2	82.8 ± 7.2		

Beads Control	79.1 ± 7.8	80.1 ± 7.4		

Table 6 shows the mean percentage scores for each possible basic behaviour as well as the mean percentage B scores, based on the behaviours Grasps, Holds and Releases, for both the children with CP and the healthy children. The behaviours Grasps, Holds and Releases were scored in almost all subtasks of the muffin and beads tasks performed by the healthy children. In contrast, these behaviours, as well as most other behaviours, were observed more often in the beads task than in the muffin task in the children with CP. The differences in percentage B-scores between the muffin and beads task were greater in the children with CP than in the healthy children (age group 1, Z = -3.684, p < 0.001; age group 2, Z = -2.295, p < 0.05).

**Table 6:** Mean percentages of each possible observed behaviour and mean percentage B-scores for both the muffin and the beads task in children with CP and in healthy children

	Healthy children, n = 56 non-dominant arm and hand				Children with CP, n = 20 affected arm and hand			
**Behaviour**	**Group 1** **Muffin** **%**	**Group 1** **Beads** **%**	**Group 2** **Muffin** **%**	**Group 2** **Beads** **%**	**Group 1** **Muffin** **%**	**Group 1** **Beads** **%**	**Group 2** **Muffin** **%**	**Group 2** **Beads** **%**

Reaches	57	67	46	86	16	32	14	22

Grasps	96	100	98	100	51	90	58	78

Holds	96	100	98	100	51	90	56	78

Releases	93	99	98	100	50	90	53	75

Stabilizes	27	67	36	76	54	92	70	87

Puts on place	59	46	44	60	10	8	3	7

Shakes, pours	20	21	12	17	6	7	8	22

Pushes, pulls, shoves, strikes	14	65	12	82	13	28	14	72

Catches, carries	17	6	26	16	3	5	3	2

Manipulates	5	3	10	7	0	2	0	2

B-score *	95	100	98	100	51	90	55	77

* obtained from the muffin task or the beads task (basic Grasps, Holds and Releases observations)

## Discussion

The results of this study show an excellent interrater, intrarater and test-retest reliability for the various outcomes of the VOAA-DDD in children with spastic unilateral cerebral palsy between 2.5 and 8 years of age. Possible factors that might have contributed to this result are the use of standardized videotapes, a computer-supported scoring system and the thorough training of the raters. Indeed, the VOAA-DDD is primarily meant to be used by pediatric occupational therapists working with children with CP. As with other occupational assessment instruments, adequate training is a prerequisite for reliable clinical application. Until now, no reliable assessment of duration and frequency of spontaneous use of the affected arm and hand was available for these children. To assess children with CP comprehensively, a range of tools is required, some of which are better suited for clinical use and others for research[14]. The selection of tools should be tailored to the clinical setting, the children involved, and the purpose of measurement.

With the VOAA-DDD two aspects of use of the affected arm and hand are scored. The results of this study indicate that both the duration of spontaneous use and the frequency scores regarding basic behaviours of the affected arm and hand can be used to determine developmental disregard, by comparing a task that merely stimulates the use of both hands with a task that demands bimanual activity. In combination with other tests like the Melbourne, the AHA and the passive and active range of motion, the VOAA-DDD gives a good overview of individual capacity and actual performance of the affected arm and hand in children with CP. Such an overview can provide indications for individually tailored rehabilitation. For instance, when a child has full active range of motion in the affected wrist and, at the same time, a low A-score on the beads task of the VOAA-DDD, it should be trained to use its capacity of wrist extension in play and daily life activities. When a child with active wrist extension would only show affected hand use during the beads but not during the muffin task of the VOAA-DDD, it should be trained to overcome its developmental disregard, e.g. by means of constraint induced movement therapy. Although one could argue that the VOAA-DDD in its present form (using a standardized situation) is not truly a test of individual performance in a 'natural' environment according to ICF definitions (ICF, WHO 2009), we believe that its task design and focus on spontaneous behaviour uniquely contribute to currently available assessment tools that mainly focus on a child's capacity.

The validity of a new assessment tool is usually determined by criterion-referenced validity and by construct validity[15]. Because there is no criterion standard for the observed duration and frequency measures available in the literature, construct validity was determined by comparing children with CP to healthy children of the same age. It was hypothesized that, in healthy children, the duration of use of both hands would be the same while stringing beads as while decorating a muffin, whereas CP children would be inclined to use their affected arm and hand significantly less during the muffin task. Because the results of this study confirmed this hypothesis, they indicate a good construct validity of the VOAA for determining developmental disregard. When using the extreme values of the healthy children as cut-off values, 90% of the CP children aged 2.5-5 years and 50% of the CP children aged 5-8 years could be identified as suffering from developmental disregard. The number of healthy children included in this study is, however, too low to determine definitive cut-off values for developmental disregard for the various age groups.

We found that it was easy to obtain adequate cooperation of the preschoolers in our study, even though preschool age is a notoriously difficult age to assess children[16]. Apparently, the VOAA-DDD tasks are child friendly and playful. The time taken to administer the VOAA-DDD in this study varied from 20 to 40 minutes, which ensures its feasibility in clinical practice. The execution of the VOAA-DDD requires knowledge of the manual, handling of two equipment boxes, and attending a 3-hours training course for learning to use the software. Some additional practice in rating videotapes to improve one's intrarater reliability is recommended. The software is available in several versions, for instance, with and without the option to simultaneously observe from two different viewing angles.

A limitation of this study is the still moderate number of children with CP that were included and the subsequent stratification in only two age groups. Nevertheless, the included children showed a nice variation of Melbourne scores indicating sufficient variability in their affected arm and hand capacity. As already mentioned, the VOAA-DDD requires a standardized test situation, whereas one would preferably assess spontaneous use of the affected arm and hand in a completely natural environment. This latter option would, however, make it much more difficult to accurately and reliably compare the execution of two specific tasks to identify developmental disregard. Further investigation is necessary to assess the responsiveness of the VOAA-DDD in therapeutic trials targeting developmental disregard as well as to determine its clinimetric properties in children older than 8 years of age.

## Conclusion

The VOAA-DDD is a reliable and valid instrument to assess spontaneous use of the affected arm and hand and to determine developmental disregard in children with spastic unilateral CP.

## Competing interests

The authors declare that they have no competing interests.

## Authors' contributions

This work was conducted as part of a PhD project of the first author (PBA), supervised by PHJ and ACG and assisted by YAG. All authors contributed to the study design, analysis and interpretation of the data, as well as to the writing of the manuscript. All authors read and approved the final manuscript.

## Pre-publication history

The pre-publication history for this paper can be accessed here:

http://www.biomedcentral.com/1471-2474/10/145/prepub
